# Orientation relationships, orientational variants and the embedding approach

**DOI:** 10.1107/S1600576723003187

**Published:** 2023-05-12

**Authors:** Richard Arnold, Peter Jupp, Helmut Schaeben

**Affiliations:** a School of Mathematics and Statistics, Victoria University of Wellington, PO Box 600, Wellington, New Zealand; b School of Mathematics and Statistics, University of St Andrews, North Haugh, St Andrews, Fife, KY16 9SS, United Kingdom; c Geophysics and Geoscience Informatics, TU Bergakademie Freiberg, Freiberg, Germany; Universität Hamburg, Germany

**Keywords:** orientation relationships, variants, determination, reconstruction, directional statistics

## Abstract

For phase transformations within polycrystalline materials, the connection between the crystal orientations of parent grains and those of child grains is usually expressed in terms of (theoretical or measured) orientation relationships. This paper introduces a new approach to various problems associated with orientation relationships: (i) estimation, (ii) whether or not a single orientation relationship fits the data adequately, (iii) whether or not a set of children comes from a common parent, and (iv) reconstruction of a parent or of grain boundaries. The approach is an extension to the crystallographic context of the well established embedding approach to directional statistics. It is inherently statistical, producing precise probabilistic statements. Explicit coordinate systems are not used and arbitrary thresholds are avoided.

## Abstract 2

A statistical approach is developed for handling some standard problems involving orientation relationships. The approach is based on directional statistics.

## 1. Introduction

Phase transformations within polycrystalline materials often induce a transformation of the texture, *i.e.* of the statistical and spatial distribution of the crystallographic orientations of the crystallites. This usually involves the replacement of each parent crystal by several child crystals, possibly in a different symmetry group. Several mathematical models for describing these transformations have been constructed on theoretical grounds. Such models include the orientation relationships of Kurdjumow–Sachs (Kurdjumow & Sachs, 1930 ▸), Nishiyama–Wassermann (Wassermann, 1933 ▸, 1935 ▸; Nishiyama,1934 ▸) and Pitsch (1959 ▸, 1967 ▸). The presence of two symmetry groups leads to the problem of multiplicity of *variants*, *i.e.* orientations of (unseen) parent crystals giving rise to orientations of given child crystals. (A precise mathematical definition is given in Section 2.3.) Much work has been carried out developing methods for fitting these models and examining their adequacy, notably by Humbert and co-authors (*e.g.* Humbert *et al.*, 1994 ▸, 1995 ▸, 2015 ▸) and by Cayron and his collaborators (*e.g.* Cayron *et al.*, 2006 ▸; Cayron, 2019 ▸). Even within the topic of the austenite–martensite transformation there are very many papers in the materials science literature on problems involving variants, *e.g.* Nolze (2004*b* ▸), Kitahara *et al.* (2006 ▸), Miyamoto *et al.* (2009 ▸), Abbasi *et al.* (2012 ▸, 2014 ▸), Koumatos & Muehlemann (2017 ▸) and Nyyssönen *et al.* (2016 ▸, 2018 ▸). These problems have been addressed by Mainprice *et al.* (1990 ▸) and Morales *et al.* (2018 ▸), for instance, in a geological context.

As far as we are aware, previous work on fitting orientation relationships and identifying variants takes the traditional viewpoint of estimating the relationship and assessing its fit, either informally or by making use of an arbitrary threshold of some measure of goodness of fit. For example, Nolze (2004*a* ▸,*b* ▸, 2005 ▸) measures the discrepancy between estimated and theoretical versions of a parameter [**A**]_1,2_ of an orientation relationship [see equation (1) below] by the (minimal) misorientation angle between representative rotations. The discrepancy is considered significant if it is larger than the accuracy of the experimental apparatus. In this paper we establish a sound statistical viewpoint on various types of problem involving orientation relationships and variants. In the traditional viewpoint, the key idea is that of being ‘numerically close’ and the typical question is ‘how far apart are the measured and theoretical quantities?’. From the statistical viewpoint, the key idea is that of being ‘statistically close’ (in a precise probabilistic sense) and the typical question is ‘what is the probability (under some hypothesis) that the measured and theoretical quantities are (at least) this far apart?’. If this probability is small then the hypothesis is rejected. We implement the statistical viewpoint here by using the embedding approach to crystallographic orientations introduced by Arnold *et al.* (2018 ▸), which is an application of the embedding approach to directional statistics [see *e.g.* Sections 2.1 and 9.1 of Mardia & Jupp (2000 ▸)]. The necessary measures of distance are constructed using orientation representations which incorporate the relevant symmetries, reducing the need to search for optimal representations within equivalence classes. The use of explicit coordinate systems is avoided.

The key points of our approach are as follows:

(i) Regarding each object such as a crystallographic orientation as a single object (equivalence class) in a quotient space, rather than considering a single representative orientation.

(ii) Avoiding arbitrary thresholds.

(iii) Avoiding arbitrarily chosen explicit coordinate systems (such as are used in asymmetric domains).

The notation used in this article is more mathematical and general than that traditionally used for discussing variants. The reasons for this choice are (i) the concepts of orientation relationship and variant are appropriate in contexts wider than the crystallographic, and (ii) for the embedding approach our notation is particularly convenient. We use the word ‘random’ in the statistical sense of ‘not deterministic’, so that repeating measurement of *e.g.* rotations will not produce exactly the same values (rather than implying that these rotations are uniformly distributed).

Some of the material in this paper has been presented in a more mathematical form suitable for statisticians by Arnold & Jupp (2019 ▸) and Arnold *et al.* (2018 ▸, 2021 ▸).

Section 2 gives a mathematical description of orientation relationships and variants. The embedding approach to crystallographic orientations is recalled in Section 3 and extended to the context of variants. Sections 4, 5, 6 and 7 treat methods for estimation (sometimes referred to as ‘calculation’, ‘determination’ or ‘evaluation’) of orientation relationships, assessment of the adequacy of a single orientation relationship, assessment of the common parentage of child crystals and reconstruction of parents, respectively. In each of these sections established methods are recalled and then methods based on the embedding approach are given. Some practical illustrations of these new methods are given in Section 8.

## 2. Orientation relationships and variants

### 2.1. Orientations of symmetrical objects

The orientation of a rigid object in 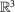 can be described by a rotation that transforms it into some standard orientation. If the object is asymmetrical then this rotation is unique, so that the orientations of the object correspond to elements of the group *SO*(3) of rotations of 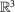 . If the object has symmetry group *K* then a rotation **U** has the same effect as the rotation **U** **H** for any **H** in *K*. Then the orientations of the object correspond to elements of the space *SO*(3)/*K*, *i.e.* the set of equivalence classes of elements of *SO*(3) under the right action of *K*. For **U** in *SO*(3) we shall denote the corresponding left coset of *K*, *i.e.* the equivalence class {**U** **H** : **H** ∈ *K*} of **U** in *SO*(3)/*K* by [**U**].

### 2.2. Orientation relationships

In many contexts, *e.g.* transformations involving a phase change, interest lies in the relationship between two random orientations with possibly different symmetry groups. Let [**U**]_1_ and [**V**]_2_ be random orientations in *SO*(3)/*K*_1_ and *SO*(3)/*K*_2_, respectively, where *K*_1_ and *K*_2_ are the symmetry groups. Particularly simple models relating [**U**]_1_ and [**V**]_2_ are the *orientation relationships.* These have the form 

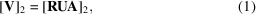

where **R** and **A** are in *SO*(3). {Note that, in general, equation (1) is not equivalent to [**U**]_1_ = [**R** ^−1^ **V** **A** ^−1^]_1_.} The rotation **R** in (1) can arise as the result of measurements being made in different coordinate systems. For example, this situation arises when the two samples are aligned at different angles to a common laboratory measurement frame. A further complication may occur if pairs of measurements ([**U**_1_]_1_, [**V**_1_]_2_),…, ([**U**_*n*_]_1_, [**V**_*n*_]_2_) are made at widely differing locations, in which case realistic modelling may require the use of an **R** that depends on location. Equation (1) determines the rotation **R** uniquely but it does not determine **A**. Indeed, because [**U**]_1_ = [**U** **H**]_1_ and [**V**]_2_ = 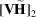 for any **H** in *K*_1_ and any 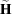 in *K*_2_, 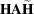 and **A** give the same orientation relationship (1). Thus (1) does not determine **A** fully but determines only its image [**A**]_1,2_ in the double coset space 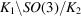 . The space 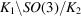 is the set of equivalence classes of elements **W** of *SO*(3) for which **W** and 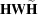 are equivalent for any **H** in *K*_1_ and any 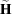 in *K*_2_. In crystallography it is usual to identify 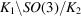 with an asymmetric domain, *i.e.* a connected subset of *SO*(3) that (apart from a set of measure zero) contains exactly one rotation in each equivalence class. Construction of asymmetric domains is considered in Section 6.3 of Morawiec (2004 ▸). Because, in any given context, there is no standard asymmetric domain, we prefer not to use such domains. An orientation relationship (1) gives rise to four types of problem:

(i) The *estimation problem* of estimating the unknown **R** and [**A**]_1,2_ on the basis of observations ([**U**_1_]_1_, [**V**_1_]_2_),…, ([**U**_*n*_]_1_, [**V**_*n*_]_2_).

(ii) The *single orientation relationship problem* of assessing whether or not a single orientation relationship can describe the data adequately.

(iii) The *sibling problem* of determining whether or not elements [**V**_1_]_2_,…, [**V**_*n*_]_2_ of *SO*(3)/*K*_2_ arise from some unknown common parent [**U**]_1_ under a known orientation relationship (**R**, [**A**]_1,2_). If [**V**_1_]_2_,…, [**V**_*n*_]_2_ are distinct then they arise from some common **U** if and only if they are part of a set of variants (see Section 2.3).

(iv) The *reconstruction problem* of estimating the unknown [**U**]_1_ on the basis of a known (**R**, [**A**]_1,2_) and observations [**V**_1_]_2_,…, [**V**_*n*_]_2_.

These problems are considered in Sections 4, 5, 6 and 7, respectively.

### 2.3. Variants

In general, an orientation relationship (1) does not yield a unique map from *SO*(3)/*K*_1_ to *SO*(3)/*K*_2_. Instead, to each [**U**]_1_ in *SO*(3)/*K*_1_ it assigns *s* distinct elements 

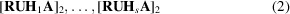

of *SO*(3)/*K*_2_, where **H**_1_,…, **H**_*s*_ ∈ *K*_1_ and 

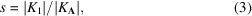


*K*_**A**_ being the *intersection group* defined as 

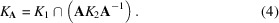

Distinctness of [**R** **U** **H**_1_ **A**]_2_,…, [**R** **U** **H**_*s*_ **A**]_2_ in (2) is equivalent to 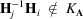 for *i* ≠ *j* (see Appendix *A* [App appa]). The [**R** **U** **H**_1_ **A**]_2_,…, [**R** **U** **H**_*s*_ **A**]_2_ in (2) are known as the (*orientation*) variants (or *crystallographic* variants) of [**U**]_1_ given by **R** and **A**. There is no distinguished variant among [**R** **U** **H**_1_ **A**]_2_,…, [**R** **U** **H**_*s*_ **A**]_2_, but the (arbitrary) choice of any one of these, [**R** **U** **H**_0_ **A**]_2_, say, as a base point determines the function 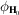 from *K*_1_ to the set of variants by 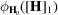 = 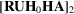 . Since 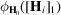 = 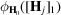 if and only if 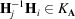 , the set (2) of variants can be identified with the coset space *K*_1_/*K*_**A**_.

As pointed out by Nolze (2008 ▸), in contrast to the commonly used theoretical orientation relationships [**A**]_1,2_, measured orientation relationships 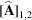 are ‘irrational’ in that their descriptions in terms of crystallographic planes and directions do not have low Miller indices. This is because it is not possible to make measurements with perfect accuracy, and so the orientations [**U**_*i*_]_1_ and [**V**_*i*_]_2_ are random. The argument in Proposition 1[Statement proposition1] in Appendix *A* [App appa] shows that the number of variants associated with a measured orientation relationship is |*K*_1_|. In real materials the variants are usually present in unequal quantities, a phenomenon known as *variant selection*, and some variants may even be absent. In many contexts it is of interest to estimate the overall proportions of the variants that are present. Whereas the orientational variants considered in this paper are cosets of *K*_**A**_ in *K*_1_, the types of variant introduced by Cayron (2016 ▸, 2019 ▸) in connection with the physical mechanisms underlying martensitic crystallography are cosets of other subgroups of *K*_1_ that describe the distortion and stretch of crystal lattices.

As pointed out by Cayron (2006 ▸), the important algebraic structure associated with the set (2) of variants is that of a groupoid, *i.e.* a set of arrows endowed with a partially defined associative composition in which every arrow has an inverse. Denote the set (2) of variants by *V*. Each triple ( 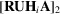 , 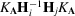 , 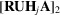 ) in 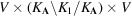 can be regarded as an arrow from [**R** **U** **H**_*i*_ **A**]_2_ to [**R** **U** **H**_*j*_ **A**]_2_. The arrows ( 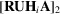 , 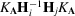 , 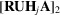 ) and ( 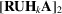 , 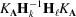 , 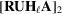 ) can be composed if and only if [**R** **U** **H**_*j*_ **A**]_2_ = [**R** **U** **H**_*k*_ **A**]_2_, in which case the composition is ( 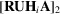 , 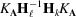 , 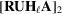 ). It is useful to combine the arrows into equivalence classes called *operators.* Each operator can be written as 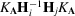 for some *i*, *j* (but, in general, *i* and *j* are not unique) and can be regarded as being a theoretical transformation that takes the variant [**R** **U** **H**_*i*_ **A**]_2_ to [**R** **U** **H**_*j*_ **A**]_2_. Thus, the set of operators can be identified with the double coset space 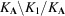 . The operator 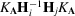 can be regarded as the misorientation between [**R** **U** **H**_*i*_ **A**]_2_ and [**R** **U** **H**_*j*_ **A**]_2_.

The composition table of the groupoid yields a multi-valued composition on the set of operators. This provides a way of attacking the sibling and reconstruction problems described in points (iii) and (iv) at the end of Section 2.2. Gey & Humbert (2003 ▸) pointed out that, in the case of the Burgers orientation relationship (*K*_1_ = *O*, *K*_2_ = *D*_6_), the number of misorientations between the variants is less than the number of variants.

## 3. The embedding approach

### 3.1. Embedding *SO*(3)/*K*

Because the coset spaces *SO*(3)/*K* are not very easy to work with, Arnold *et al.* (2018 ▸) (see also Arnold & Jupp, 2019 ▸) developed the *embedding approach* in which a function **t** : *SO*(3)/*K* → *E* is used to send *SO*(3)/*K* into (but not onto) some inner-product space *E*. The function **t** is required to be (i) one-to-one, (ii) equivariant, *i.e.* 〈**t**([**V** **U**]), **t**([**V** **W**])〉 = 〈**t**([**U**]), **t**([**W**])〉 for **U**, **V**, **W** in *SO*(3), where 〈·, ·〉 denotes the inner product, (iii) such that **t**([**U**]) has expectation 0 if [**U**] is uniformly distributed on *SO*(3)/*K*. For the crystallographic groups *C*_1_, *C*_2_, *C*_3_, *C*_4_, *D*_2_, *D*_6_, *T* and *O* some useful functions **t** are given explicitly in Table 1 ▸. Together **t** and 〈·, ·〉 lead us to 

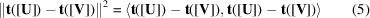

as a new measure of squared distance between elements [**U**] and [**V**] of *SO*(3)/*K*. By design it incorporates the symmetry group *K* and it replaces the need for misorientation angles. A useful summary of [**U**_1_],…, [**U**_*n*_] is their *sample mean*, 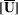 , which is defined as the element of *SO*(3)/*K* that minimizes 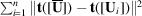 or, equivalently, maximizes 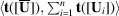 . For *K* = *C*_*r*_ or *D*_*r*_ explicit approximations to sample means are given in Section 2.3 of Arnold & Jupp (2019 ▸).

Embeddings are discussed further in Appendix *B* [App appb].

### 3.2. Embedding *K*_1_\*SO*(3)/*K*_2_

The double coset spaces 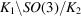 are even more complicated than the coset spaces *SO*(3)/*K*, but the embedding approach can be used for them also.

Any **t** : *SO*(3)/*K*_2_ → *E*, where *E* is a vector space, can be averaged over *K*_1_ to give a corresponding 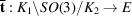 , defined by 

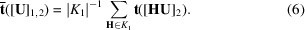

We shall exploit such 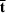 in order to carry out inference on orientation relationships. If **t** has properties (ii) and (iii) of Section 3.1 then so does 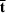 . On the other hand, **t** having property (i) (being one-to-one) does not imply that 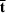 has this property also. In a few very special cases (*e.g.* *K*_1_ and *K*_2_ isomorphic to *C*_2_ and *C*_3_ and with a common axis) 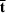 is identically zero. In the context of phase transitions it seems that such symmetries are not of practical interest.

## 4. Estimation

### 4.1. Estimation based on orientations of parents and children: established methods

Suppose that we are given paired observations ([**U**_1_]_1_, [**V**_1_]_2_),…, ([**U**_*n*_]_1_, [**V**_*n*_]_2_) with [**U**_*i*_]_1_ in *SO*(3)/*K*_1_ and [**V**_*i*_]_2_ in *SO*(3)/*K*_2_ for 1 ≤ *i* ≤ *n*. It is assumed that the pairs ([**U**_1_]_1_, [**V**_1_]_2_),…, ([**U**_*n*_]_1_, [**V**_*n*_]_2_) are observations of a pair ([**U**]_1_, [**V**]_2_) of random orientations that satisfy the orientation relationship (1). The problem is that of estimating the unknown [**A**]_1,2_ (and **R** if it is not known). In the case *K*_1_ = *K*_2_ = *C*_1_ (*i.e.* no symmetry, so that variants do not occur), an explicit estimate of **A** was given by Mackenzie (1957 ▸). In the general case, it follows from (1) that 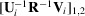 is close to [**A**]_1,2_ for *i* = 1,…, *n*. Then 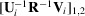 can be considered as the ‘local’ estimate of [**A**]_1,2_ given by the observed pair ([**U**_*i*_]_1_, [**V**_*i*_]_2_). Here ‘local’ is used in the sense that the estimate uses only quantities with index *i*, which in many cases means that they are measured at the *i*th location.

Using this approach Nolze found that, for transformation from face-centred cubic to body-centred cubic lattices, the standard theoretical models did not provide a good fit to the experimental data that he was investigating

### 4.2. Estimation based on orientations of parents and children: embedding approach

The embedding approach uses a suitable embedding **t**_2_ : *SO*(3)/*K*_2_ → *E*. If ([**U**_1_]_1_, [**V**_1_]_2_),…, ([**U**_*n*_]_1_, [**V**_*n*_]_2_) are observations on a pair ([**U**]_1_, [**V**]_2_) of random orientations that satisfy the orientation relationship (1) then 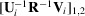 is close to [**A**]_1,2_ for *i* = 1,…, *n*. Therefore, it is sensible to estimate **R** and [**A**]_1,2_ by 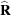 and 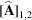 , which are the **R** and [**A**]_1,2_ that minimize the squared distance 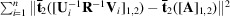 , or equivalently that maximize 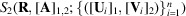 , defined by 

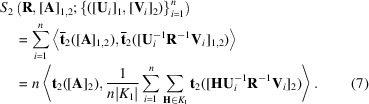

Thus, [**A**]_1,2_ can be taken as the sample mean of the images by 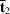 [defined by (6)] of 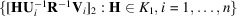 . An alternative method of estimation uses the **R** and [**A**]_1,2_ that maximize 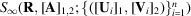 , which is defined by 

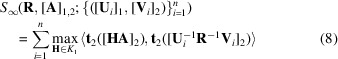

[see Section 6.1 of Arnold *et al.* (2021 ▸)].

Not only can a point estimate 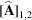 of [**A**]_1,2_ be obtained, it is also possible to get confidence regions for [**A**]_1,2_. Bootstrap confidence regions for [**A**]_1,2_ can be calculated by resampling the data as follows. For a suitable *B* and *m*, for *b* = 1,…, *B*, sample (with replacement) *m* pairs (*m* ≤ *n*) ([**U**_*b*1_]_1_, [**V**_*b*1_]_2_),…, ([**U**_*bm*_]_1_, [**V**_*bm*_]_2_) from ([**U**_1_]_1_, [**V**_1_]_2_),…, ([**U**_*n*_]_1_, [**V**_*n*_]_2_). Denote the estimate of [**A**]_1,2_ based on ([**U**_*b*1_]_1_, [**V**_*b*1_]_2_),…, ([**U**_*bm*_]_1_, [**V**_*bm*_]_2_) by 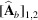 . Define the similarity measure 

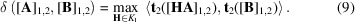

For 0 < α < 1, define *c*_α_ by 

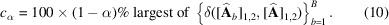

Then a 100 × (1 − α)% bootstrap confidence region for [**A**]_1,2_ is 

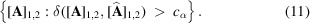

For the corresponding test of *H*_0_ : [**A**]_1,2_ = [**A**_0_]_1,2_ (where [**A**_0_]_1,2_ is a specified theoretical [**A**]_1,2_), the *p*-value (*i.e.* the probability under *H*_0_ of another sample producing a value of 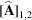 at least as extreme as that observed) is 

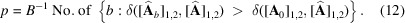




The hypothesis *H*_0_ is rejected at significance level α if *p* ≤ α or, equivalently, if the estimated 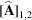 lies outside the 100 × (1 − α)% bootstrap confidence region (11) for [**A**]_1,2_.

**Figure 1:**
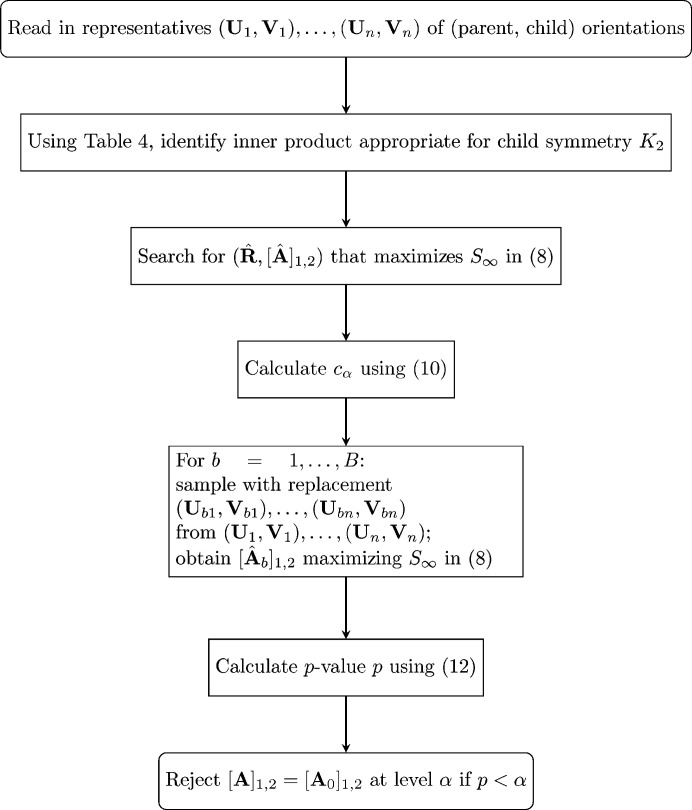
A flow chart for (i) estimation of (**R**, [**A**_1,2_]) based on (**U**_1_, **V**_1_),…, (**U**_*n*_, **V**_*n*_), (ii) confidence regions for [**A**_1,2_] and (iii) testing of [**A**_1,2_] = [**A**_0__1,2_]. An alternative method minimizes *S*_2_ in (7) instead of *S*_∞_ in (8).

A flow chart for implementation of the techniques described in this subsection is given in Fig. 1 ▸.

### 4.3. Estimation based on orientations of children alone: established methods

In some settings, no observations on the orientations of parent grains are available. Nevertheless, in many cases it is possible to estimate [**A**]_1,2_ from orientations [**V**_1_]_2_,…, [**V**_*n*_]_2_ of child grains.

The estimation method of Humbert *et al.* (2015 ▸) starts from observed child crystallographic orientations [**V**_1_]_2_,…, [**V**_3_]_2_ that are the visible part of ([**U**_1_]_1_, [**V**_1_]_2_), ([**U**_2_]_1_, [**V**_2_]_2_), ([**U**_3_]_1_, [**V**_3_]_2_). These pairs are taken to obey (at least approximately) a form of the orientation relationship (1), so that 

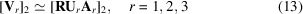

for some **U**_1_, **U**_2_, **U**_3_ and **A**_1_, **A**_2_, **A**_3_. It is assumed that **U**_1_ ≃ **U**_2_ ≃ **U**_3_ ≃ **U** and **A**_1_ ≃ **A**_2_ ≃ **A**_3_ ≃ **A** for some **U** and **A** in *SO*(3) (assumptions that are reasonable if the orientations are measured at points near a triple junction and the local orientation relationships vary only slowly with position). It then follows from (13) that for *i*, *j* in {1, 2, 3} it is possible to choose **H**_*i*_, **H**_*j*_ in *K*_1_ and 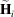 , 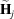 in *K*_2_ such that **U**_*i*_ ≃ **U** **H**_*i*_, **U**_*j*_ ≃ **U** **H**_*j*_ and 

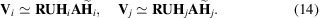

Then 

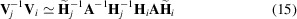

or, equivalently, 

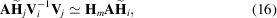

where 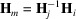 . For given *i* and *j*, solutions for **A** of the equation obtained from (16) by replacing approximate equality by exact equality are far from unique. On the other hand, for general **V**_1_, **V**_2_, **V**_3_ (outside some set of measure 0), the three corresponding equations as *i*, *j* run through distinct (unordered) pairs in {1, 2, 3} do have a unique solution. In view of this, Humbert *et al.* (2015 ▸) suggested estimating the rotation **A** by any element of *SO*(3) that minimizes 

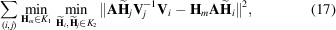

the sum being over (*i*, *j*) = (1, 2), (1, 3), (2, 3) and the norm being the Frobenius norm [= Hilbert–Schmidt norm, defined by ∥**B**∥^2^ = trace(**B** **B** ^T^)].

The estimation stage of the iterative reconstruction method of Nyyssönen *et al.* (2016 ▸, 2018 ▸) is also motivated by (16). Given (i) orientations [**V**_*i*_]_2_ and [**V**_*j*_]_2_ of distinct variants and (ii) **H**_*m*_ in *K*_1_ and 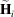 , 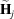 in *K*_2_, [**A**]_1,2_ is estimated by 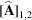 , where 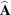 is found by iterative solution of 

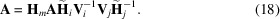

Since **H**_*m*_, 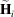 and 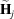 are not known, Nyyssönen and co-workers recommend averaging the above 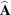 as **H**_*m*_, 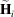 , 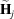 run through *K*_1_, *K*_2_, *K*_2_. Note that averaging of rotations is not well defined.

### 4.4. Estimation based on orientations of children alone: embedding approach

Given an embedding **t**_2_ : *SO*(3)/*K*_2_ → *E*, define 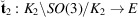 by 

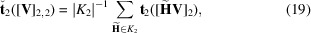

where [**V**]_2,2_ denotes the image of [**V**]_2_ in the double coset space 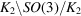 .

It follows from (15) that 

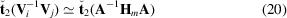

for some **H**_*m*_ in *K*_1_. It is therefore reasonable to estimate [**A**]_1,2_ as any element of 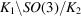 that minimizes 

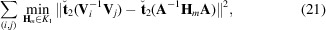

the sum being over ordered pairs (*i*, *j*) of distinct elements of {1,…, *n*}.

**Figure 2:**
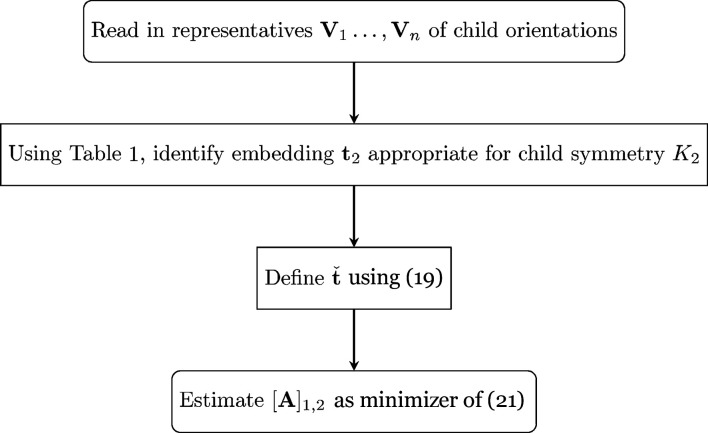
A flow chart for estimation of [**A**_1,2_] based on **V**_1_,…**V**_*n*_.

A flow chart for implementation of the technique described in this subsection is given in Fig. 2 ▸.

## 5. Assessing the adequacy of a single orientation relationship

Whereas some data sets can be fitted well by the orientation relationship (1), for others such a single orientation relationship is inadequate and several orientation relationships are required. For data sets in which a single orientation relationship does not suffice, it is possible to identify clusters of observations within each of which the data share a single orientation relationship.

### 5.1. Established methods

We are unaware of anything in the crystallographic literature that considers exactly this problem.

### 5.2. Embedding approach

One way of exploring the adequacy of (1) is to use cluster analysis to divide the *n* pairs of observations ([**U**_1_]_1_, [**V**_1_]_2_),…, ([**U**_*n*_]_1_, [**V**_*n*_]_2_) into subsets, each of which consists of pairs that give similar estimates of [**A**]_1,2_. Cluster analysis is described in detail by Everitt *et al.* (2011 ▸), who give several algorithms. We find it convenient to use the following simple divisive algorithm. For an estimate 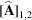 found using the method of Section 4.2 and for *i* = 1,…, *n*, define *d*_*i*_ by 

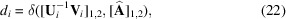

where δ is defined in (9). The maximum possible value of *d*_*i*_ is 

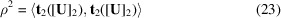

[which does not depend on **U** in *SO*(3)]. A value of *d*_*i*_ near ρ^2^ indicates that the ‘local estimate’ 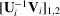 of [**A**]_1,2_ is close to the ‘global estimate’ 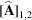 . The values of ρ^2^ corresponding to various symmetry groups are given in Table 2 ▸. If the single orientation relationship 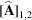 gives a good fit to the data ([**U**_1_]_1_, [**V**_1_]_2_),…, ([**U**_*n*_]_1_, [**V**_*n*_]_2_) then *d*_1_,…, *d*_*n*_ are all large. Placing all observations with *d*_*i*_ close to ρ^2^ into a cluster, re-estimate 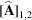 for that cluster. Repeat this process on the remaining observations, thus grouping them sequentially into clusters having similar local orientation relationships. If only one cluster is found then the single orientation relationship 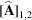 will describe the data well. Whereas the clustering of locations used by Johnstone *et al.* (2020 ▸) and Ostapovich & Trusov (2021 ▸) in the construction of crystal orientation maps takes place on *SO*(3)/*K* and is based on misorientation angles, the clustering used here takes place on 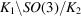 and is based on the *d*_*i*_ of (22). The clustering of locations used by Gomes de Araujo *et al.* (2021 ▸) in the reconstruction of parent microstructure is based on misorientation angles between adjacent grains.

**Figure 3:**
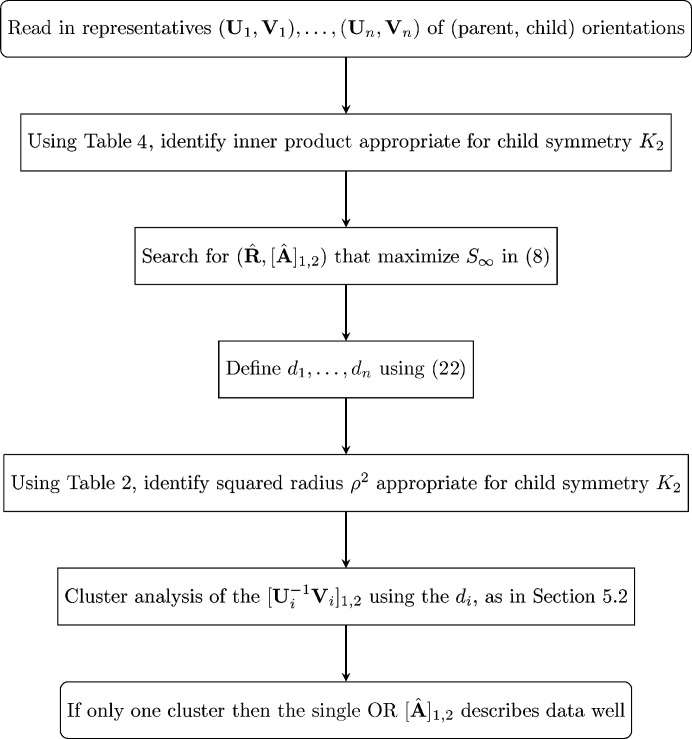
A flow chart for assessing the adequacy of a single orientation relationship (OR).

A flow chart for implementation of the technique described in this subsection is given in Fig. 3 ▸.

## 6. Common parentage

In some contexts, such as locating the boundaries of parent grains, it is useful to be able to assess whether or not a set of child crystals are from the same parent. Observed elements [**V**_1_]_2_,…, [**V**_*n*_]_2_ of *SO*(3)/*K*_2_ can be considered as arising from some common parent if and only if they are close to variants corresponding to some element of *SO*(3)/*K*_1_.

### 6.1. Established methods

The method of Gey & Humbert (2003 ▸) and Karthikeyan *et al.* (2006 ▸) assesses [**V**_1_]_2_,…, [**V**_*n*_]_2_ to be from the same parent if the observed misorientation angles between [**V**_*i*_]_2_ and [**V**_*j*_]_2_ for 1 ≤ *i*, *j* ≤ *n* are within some given threshold of the theoretical misorientation angles. These observed misorientation angles are 

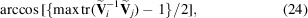

where the maximum is over 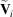 in *SO*(3) with 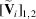 = 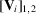 for *i* = 1,…, *n*.

Cayron *et al.* (2006 ▸) considered the problem of finding maximal sets [**V**_1_]_2_,…, [**V**_*n*_]_2_ of children from a common parent. Their method is based on considering triples [**V**_*i*_]_2_, [**V**_*j*_]_2_ and [**V**_*k*_]_2_ to be from the same parent if they are *coherent*, *i.e.* there are operators *O*_*ij*_, *O*_*ik*_, *O*_*jk*_ taking [**V**_*i*_]_2_ to [**V**_*j*_]_2_, [**V**_*i*_]_2_ to [**V**_*k*_]_2_, [**V**_*j*_]_2_ to [**V**_*k*_]_2_, respectively, (at least approximately) and with *O*_*ik*_ as some value of the composition of *O*_*jk*_ with *O*_*ij*_. Such a ‘nucleus’ triple is ‘grown’ by adding progressively further grains, to obtain a set of grains in which each triple is coherent. This is continued until a maximal such set is obtained.

### 6.2. Embedding approach

The embedding approach uses a suitable embedding **t**_2_ : *SO*(3)/*K*_2_ → *E*. Divisive cluster analysis is then applied to **t**_2_([**V**_1_]_2_),…, **t**_2_([**V**_*n*_]_2_) using 

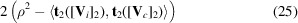

as a measure of squared distance [see (5)] between [**V**_*i*_]_2_ and the putative centre [**V**_*c*_]_2_ of a cluster. The [**V**_*c*_]_2_ for a cluster is chosen to minimize the sum of squared distances from that centre to the members of the cluster (*i.e.* it is set to be the sample mean defined in Section 3.1). If only one cluster is found then it is considered that [**V**_1_]_2_,…, [**V**_*n*_]_2_ arise from the same parent.

**Figure 4:**
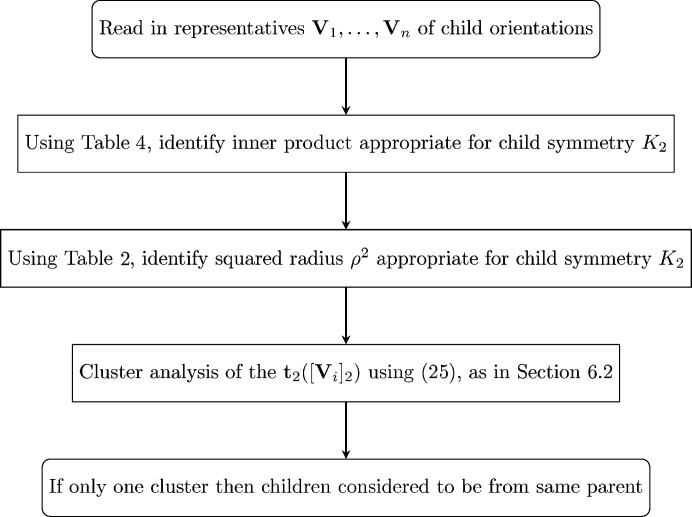
A flow chart for assessing common parentage.

A flow chart for implementation of the technique described in this subsection is given in Fig. 4 ▸.

## 7. Reconstruction

In some contexts the orientations [**V**_1_]_2_,…, [**V**_*n*_]_2_ of child crystals are observed but the parent crystals are not visible. The *reconstruction problem* is that of estimating the orientation [**U**]_1_ of the parent crystal, assuming a given orientational relationship of the form (1) with **R** and [**A**]_1,2_ known. Thus, the problem is to estimate [**U**]_1_ such that [**V**_*i*_]_2_ is close to [**R** **W**_*i*_ **A**]_2_ (*i* = 1,…, *n*) for some **W**_1_,…, **W**_*n*_ with [**W**_1_]_1_ = ⋯ = [**W**_*n*_]_1_ = [**U**]_1_.

### 7.1. Established methods

The reconstruction method introduced by Humbert *et al.* (1994 ▸) is based on the fact that, if the pairs ([**U**]_1_, [**V**_*i*_]_2_) and ([**U**]_1_, [**V**_*j*_]_2_) each satisfy the orientation relationship (1) (at least approximately) then (14) holds. It follows that 

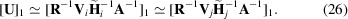

If **R** and **A** are known then applying this method to the three pairs (*i*, *j*) obtained from three variants yields a unique value for [**U**]_1_. If observations on *n* variants are available then Humbert & Gey (2002 ▸) and Gey & Humbert (2003 ▸) recommend that the values of [**U**]_1_ given by the 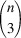 triples of variants be averaged. Note that averaging of rotations is not well defined.

### 7.2. Embedding approach

One reconstruction method using the embedding approach starts with a suitable embedding **t**_2_ of *SO*(3)/*K*_2_ and known (or estimated) values of **R** and [**A**]_2_. It then calculates **W**_1_,…, **W**_*n*_ by the 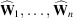 that maximize 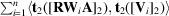 , *i.e.* that maximize 

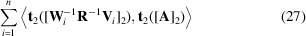

over **W**_1_,…, **W**_*n*_ satisfying [**W**_1_]_1_ = ⋯ = [**W**_*n*_]_1_, and then estimates [**U**]_1_ by 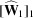 . In other words, the estimate of [**U**]_1_ is the element of *SO*(3)/*K*_1_ corresponding to the **U** in *SO*(3) that maximizes 

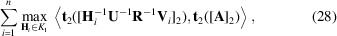

where **A** is any representative in *SO*(3) of [**A**]_1,2_. In (28) it can be assumed without loss of generality that **H**_1_ = **I**_3_, the identity.

Locating the maximum of (28) involves maximization over 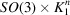 , so it may be useful to consider an alternative estimator which is easier to compute. The left-hand approximate equality in (14) gives 

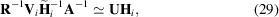

and so 

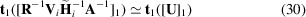

for any embedding **t**_1_ of *SO*(3)/*K*_1_. Define 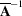 by 

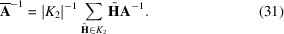

Then (30) gives 

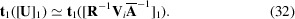

Thus it is reasonable to estimate [**U**]_1_ by the sample mean (based on **t**_1_) of 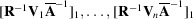 , *i.e.* the [**U**]_1_ that maximizes 

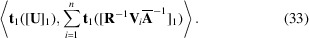




**Figure 5:**
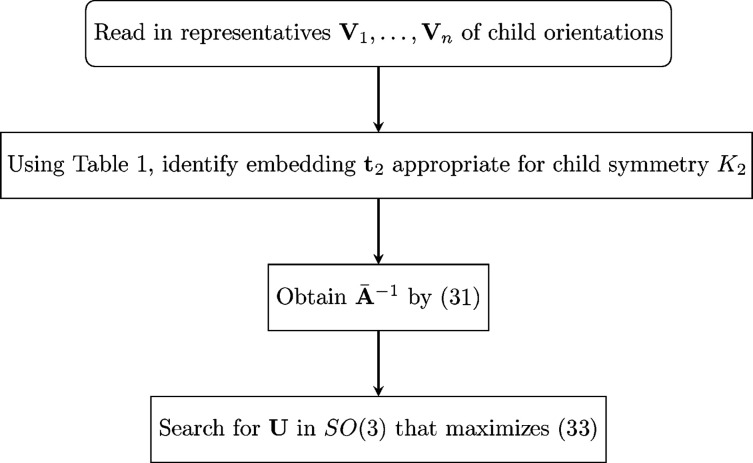
A flow chart for reconstruction of a parent.

A flow chart for implementation of the technique described in this subsection is given in Fig. 5 ▸.

## 8. Practical applications

### 8.1. Spatially varying orientation relationship: austenite–martensite transformation

**Figure 6:**
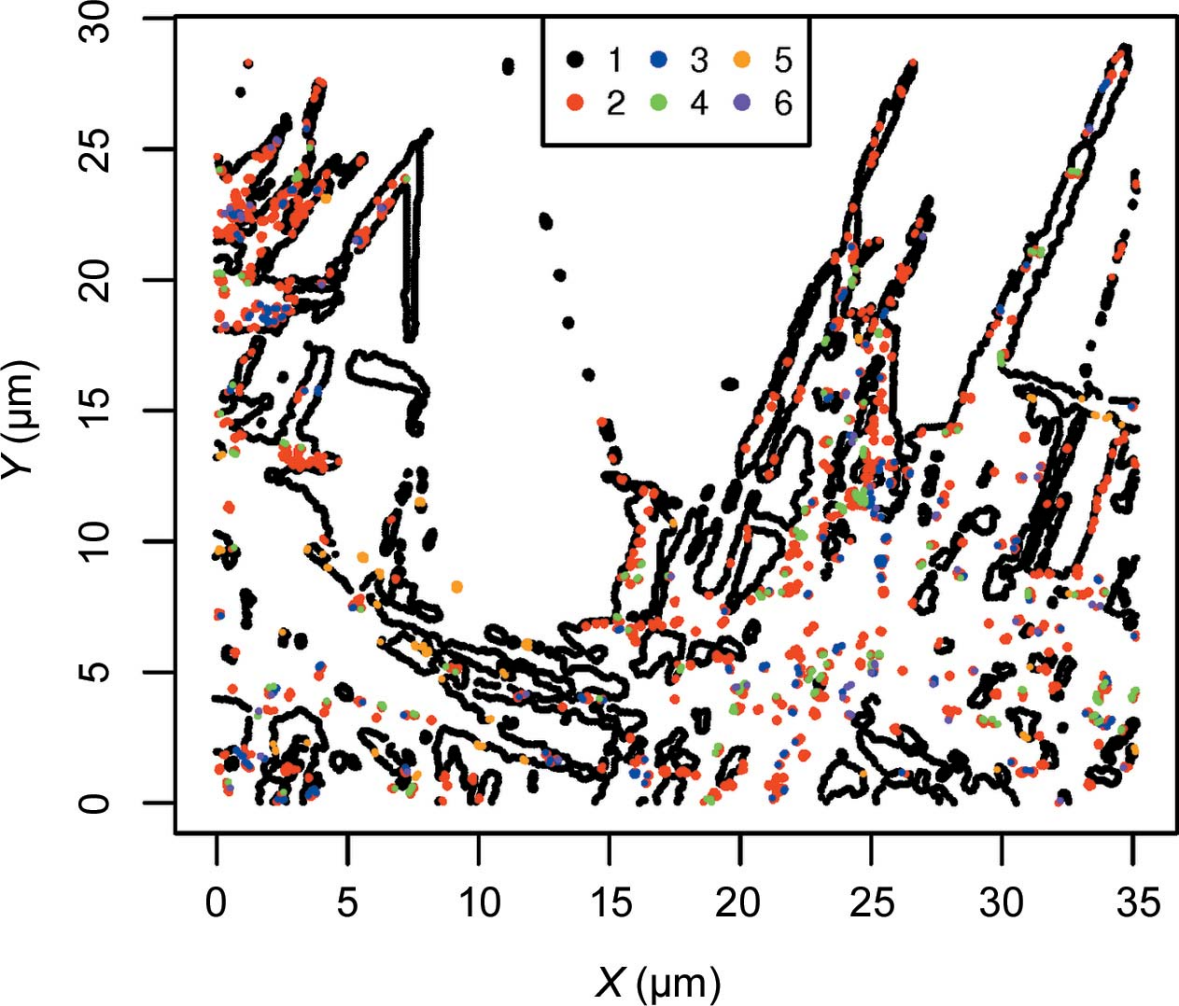
Locations of sites coloured by cluster.

**Figure 7:**
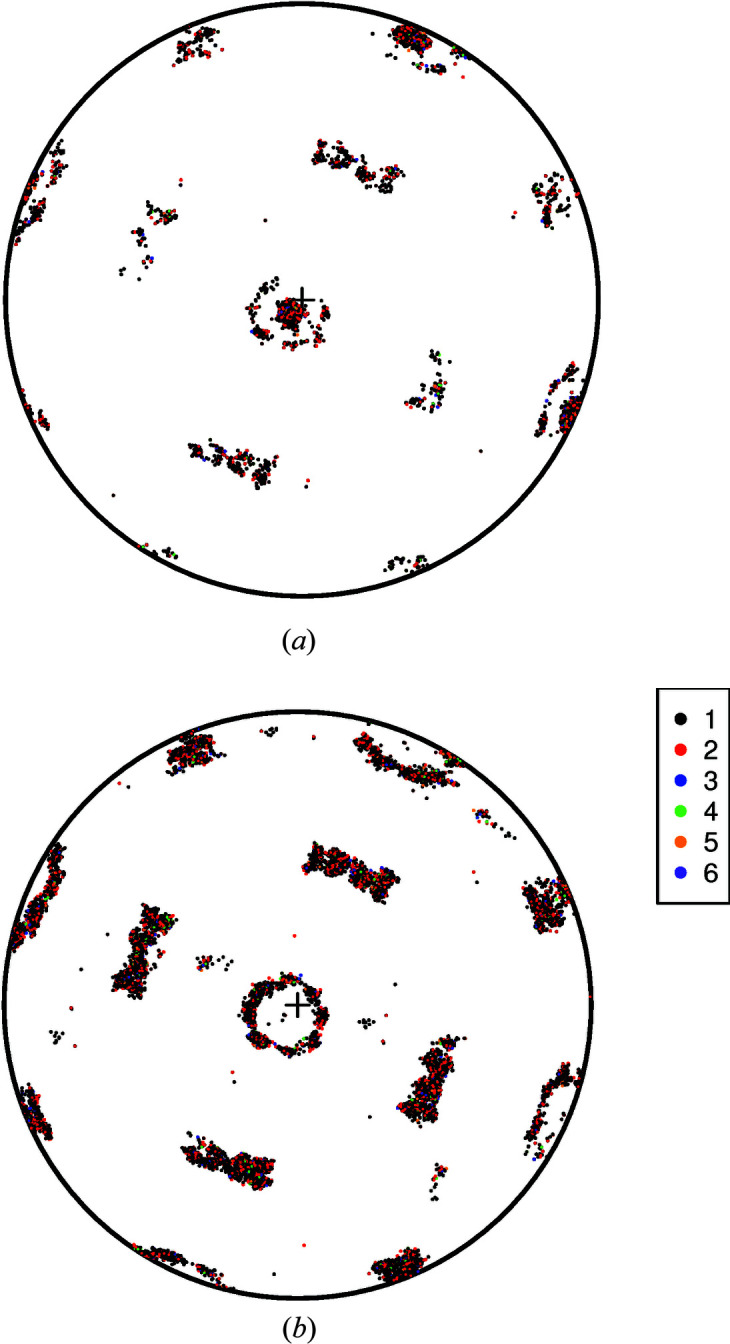
(*a*) A stereonet diagram showing the *n* = 9707 orientations [**U**_*i*_]_1_, *i* = 1,…, *n*, in the austenite phase from Wendler *et al.* (2017 ▸). (*b*) The corresponding martensite orientations [**V**_*i*_]_2_, *i* = 1,…, *n*.

The data set considered by Wendler *et al.* (2017 ▸) consists of 9707 pairs of orientations ([**U**_*i*_]_1_, [**V**_*i*_]_2_), *i* = 1,…, 9707, of austenite and martensite, respectively. For each pair the orientations are measured at sites that are close but separated by a boundary between grains. The locations of the pairs on the surface of a steel sample are shown in Fig. 6 ▸. Fig. 7 ▸ displays these orientations on two stereonets (stereographic projections), one for the (face-centred cubic) austenite phase and the other for the (body-centred cubic) martensite phase. The symmetry groups *K*_1_ and *K*_2_ are both equal to the octahedral group *O*. In Fig. 7 ▸, each disc represents the upper half of the unit sphere, and each orientation [**U**_*i*_]_1_ or [**V**_*i*_]_2_ is represented by the three points at which the three (unordered) orthogonal axes determined by [**U**_*i*_]_1_ or [**V**_*i*_]_2_ intersect the upper half of the unit sphere. In each diagram there is considerable variation, which is due partly to the differing crystal orientations [**U**_*i*_]_1_ of the austenite phase prior to the transformation. However, there is a degree of clustering present, which can be explained by differing orientation relationships [**A**]_1,2_ among the pairs of observations.

A cluster analysis, as outlined in Section 5, using *S*_∞_ from (8) [rather than *S*_2_ from (7)] was carried out. It reveals six clusters in 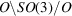 of values 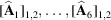 of 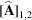 . Most observations belong to cluster 1 (*n*_1_ = 7214) or cluster 2 (*n*_2_ = 1844). Note that this is not a spatial clustering but rather a clustering of common orientation relationships. The locations of the clusters are shown by the colouring of the symbols in Fig. 6 ▸, and all six clusters occur at locations spread all over the surface of the steel sample.

**Figure 8:**
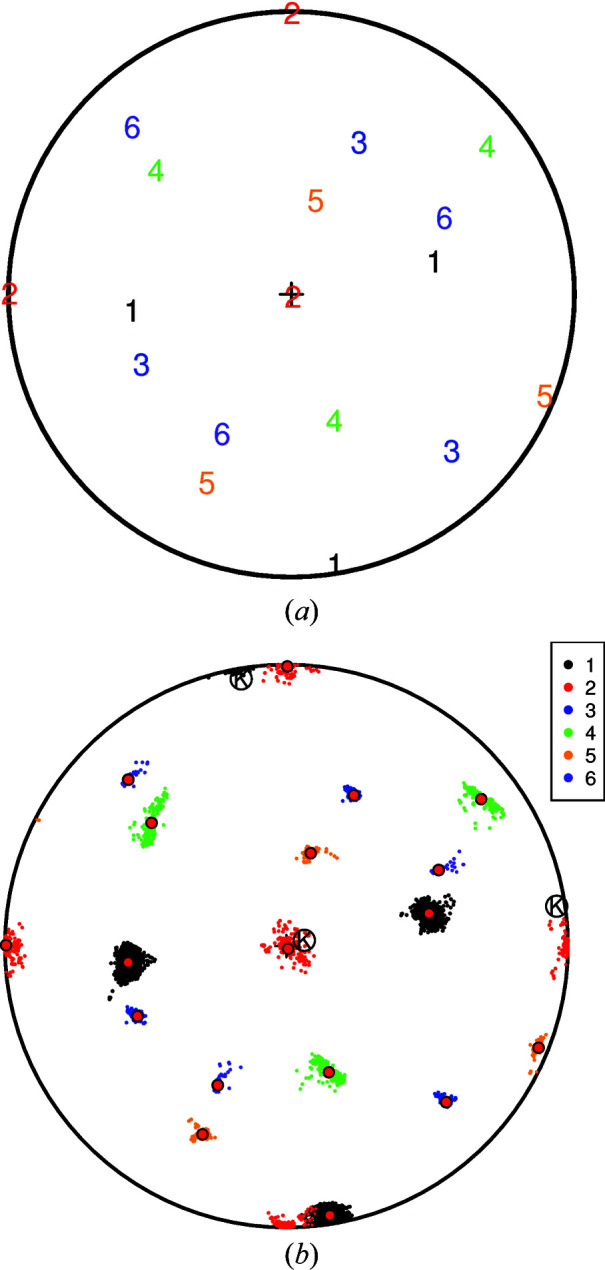
(*a*) Orientation relationships 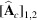 in the six fitted clusters *c* = 1,…, 6. The orientations are plotted as members of *SO*(3)/*K*_2_ by (arbitrarily) selecting the element of *SO*(3)/*O* for which 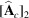 is closest to the first observation in each cluster: 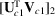 . (*b*) Under the same convention as in panel (*a*), the data 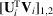 are shown, coloured by cluster. The fitted orientations 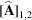 are shown as red filled circles.

Fig. 8 ▸(*a*) shows the estimated orientations 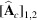 for each of the six clusters, *c* = 1,…, 6. These same orientations are shown together with the 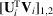 in Fig. 8 ▸(*b*). In drawing diagrams of this type we have had to make the choice to display each 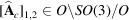 as an element 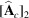 of *SO*(3)/*O*, (arbitrarily) selecting the representation closest to the first observation 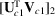 in each cluster.

Fig. 7 ▸(*b*) appears to be a left–right reflection of the centre plot [labelled ‘{100}’) in Fig. 5(*d*) of Wendler *et al.* (2017 ▸)]. (That the figures are not identical may be due to a difference in convention regarding Euler angles.) The colour codings in the two figures are completely different; that in Fig. 7 ▸(*b*) is derived from having taken full account of all ambiguities in rotation.

### 8.2. Testing whether [**A**]_1,2_ has a given value

**Figure 9:**
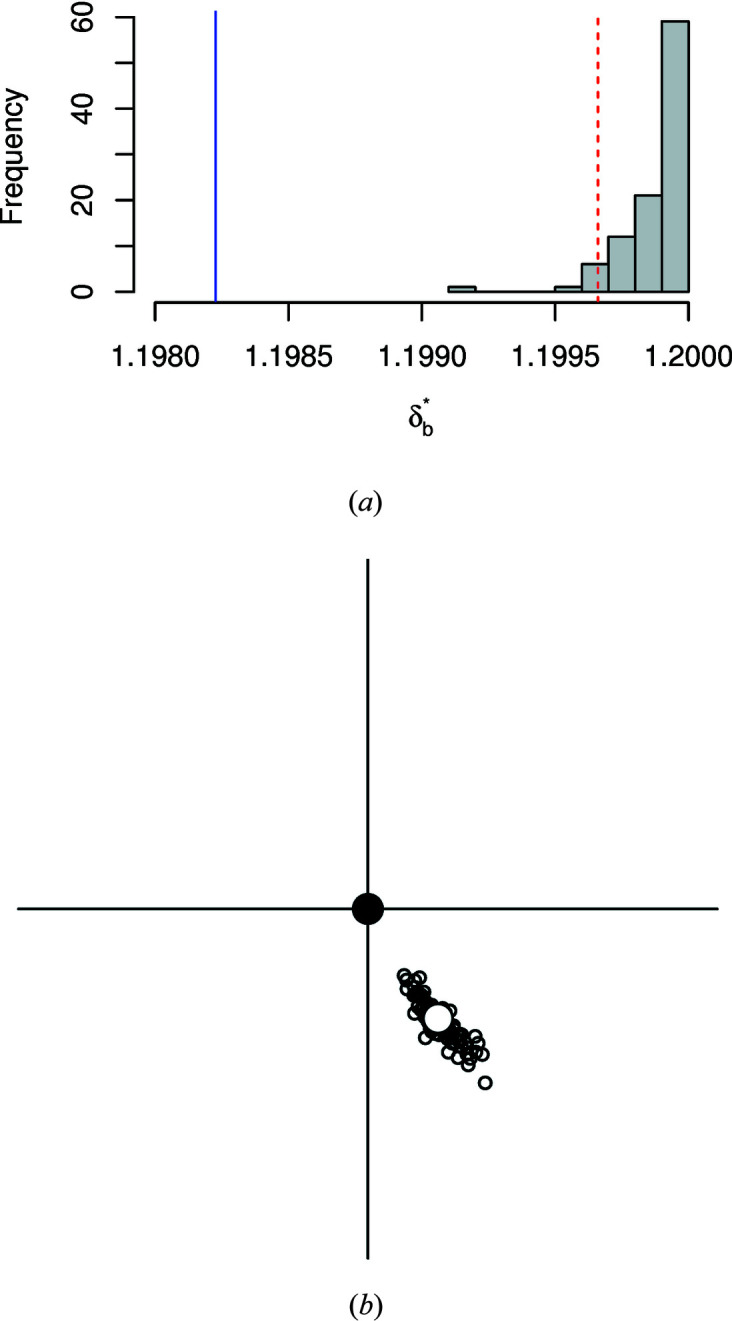
(*a*) A histogram of bootstrap 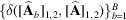 values. The limit *c*_α_ of the 95% confidence region is indicated by the red dashed line. The estimated δ value for the hypothesized value is the vertical solid blue line on the left-hand side. (*b*) Locations of bootstrap estimates on an enlarged stereonet. The estimate 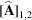 is shown as the large open circle, the hypothesized value [**I**_3_]_1,2_ as a filled circle at the origin, and the bootstrap estimates as small open circles. The large cross in (*b*) is the same size as the crosses at the centres of the stereonets in Figs. 7 ▸ and 8 ▸.

In the analysis in Section 8.1 the estimated orientation relationship 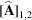 for cluster 2 is very close to the identity **I**_3_. We can test the hypothesis *H*_0_ : [**A**]_1,2_ = [**I**_3_]_1,2_ by forming a bootstrap confidence region for 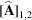 using the methods of Section 4. For demonstration purposes, we took a random subset of 50 observations from cluster 2. We then generated *B* = 100 bootstrap samples from this subset, and re-estimated [**A**]_1,2_ in each case. The distribution of values of 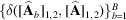 is shown in Fig. 9 ▸(*a*), and the similarity measure δ_0_ for the hypothesized value falls well outside the distribution, a convincing rejection of *H*_0_. Fig. 9 ▸(*b*) displays the original estimate 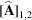 and the 100 replicate estimates, all lying well away from the hypothesized value [**I**_3_]_1,2_.

## 9. Software

The analyses in Section 8 were performed using the freely available statistical software *R* (https://www.R-project.org/). A general and flexible MATLAB tool kit for the analysis of crystallographic data is provided by the open-source crystallographic toolbox *MTEX* (Bachmann *et al.*, 2010 ▸).

## 10. Conclusions

The intrinsic symmetries of crystal structures provide a challenge to statistical analysis due to the ambiguities of their representations. The challenge becomes greater in a setting where the orientations of crystals with different symmetries are to be compared.

In this paper we have applied the embedding approach from directional statistics to the representation of crystallographic orientation data. This approach enables us to reformulate standard problems of estimation and inference in the crystallographic setting, eliminating the ambiguities which arise from the crystallographic symmetries, while retaining the genuine multiplicity of crystallographic variants.

Although the embedded objects we work with may be unfamiliar, they are straightforward to implement in software, and should provide a practical tool for researchers seeking to characterize crystallographic structures and their transformations.

## Figures and Tables

**Table 1:** Some embeddings **t**_*K*_ : *SO*(3)/*K* → *E* For *C_r_* with *r* ≥ 3, **u**_0_ = [sin(2π/*r*)]^−1^ **u**_1_ × **u**_2_. For *D*_2_, **u**_3_ = ±**u**_1_ × **u**_2_. The symmetric arrays ⊗^*r*^ **u**_*i*_ and **N**_*r*_ are defined in equations (37) and (38), respectively.

Group, *K*	**t**_*K*_
*C*_1_	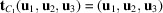
*C*_2_	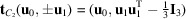
*C*_3_	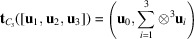
*C*_4_	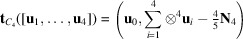
*D*_2_	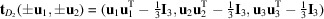
*D*_4_	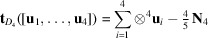
*D*_6_	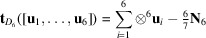
*T*	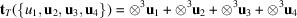
*O*	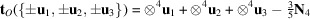

**Table 2:** Values of the squared radius ρ^2^ ρ^2^ is defined in equation (23).

Group, *K*	ρ^2^
*C*_1_	3
*C*_2_	5/3
*C_r_* (*r* ≥ 3)	
*r* odd	1 + 2^1−*r*^ *r* ^2^
*r* even	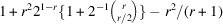
*D*_2_	2
*D_r_* (*r* ≥ 3)	
*r* odd	2^1−*r*^ *r* ^2^
*r* even	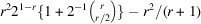
*T*	32/9
*O*	6/5

**Table 3:** Symmetry groups and frames The **u**_*i*_ are unit vectors. |*K*| is the number of elements in *K*.

Group, *K*	Name	|*K*|	Frame	Notes
*C*_1_	Trivial	1	(**u**_1_, **u**_2_, **u**_3_)	**u**_1_, **u**_2_, **u**_3_ orthonormal, **u**_3_ = **u**_1_ × **u**_2_
*C*_2_	Cyclic	2	(**u**_0_, ± **u**_1_)	**u**_0_, **u**_1_ orthonormal
*C_r_* (*r* ≥ 3)	Cyclic	*r*	(**u**_1_,…, **u**_*r*_)	**u**_1_,…, **u**_*r*_ coplanar, known up to cyclic order, 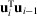 = cos(2π/*r*) for *i* = 2,…, *r*
*D*_2_	Dihedral	4	(±**u**_1_, ± **u**_2_)	Orthogonal axes
*D*_6_	Dihedral	12	(**u**_1_,…, **u**_6_)	**u**_1_,…, **u**_6_ coplanar, known up to cyclic order and reversal, 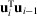 = cos(π/3) for *i* = 2,…, 6
*T*	Tetrahedral	12	{**u**_1_,…, **u**_4_}	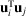 = −1/3 for *i* ≠ *j*
*O*	Octahedral = cubic	24	{±**u**_1_, ± **u**_2_, ± **u**_3_}	Orthogonal axes

**Table 4:** Inner products of transforms of symmetric frames For *C_r_* with *r* ≥ 3, **u**_0_ = {sin(2π/*r*)}^−1^ **u**_1_ × **u**_2_. For *D*_2_, **u**_3_ = ±**u**_1_ × **u**_2_.

Group, *K*	Inner product
*C*_1_	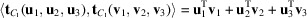
*C*_2_	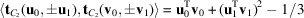
*C*_3_	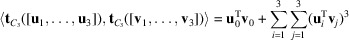
*C*_4_	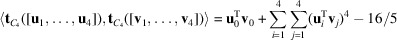
*D*_2_	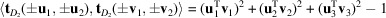
*D*_4_	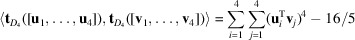
*D*_6_	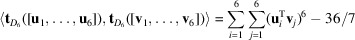
*T*	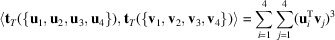
*O*	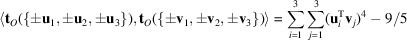

## Appendix A The number of variants

In the context of Section 2.3, elements [**U** **H**_*i*_]_1_ and [**U** **H**_*j*_]_1_ of *SO*(3)/*K*_1_ give rise to the same element of *SO*(3)/*K*_2_ under orientation relationship (1) if and only if [**R** **U** **H**_*i*_ **A**]_2_ = [**R** **U** **H**_*j*_ **A**]_2_. Since

the number of variants is *s* = |*K*_1_|/|*K*_**A**_|.

Proposition 1 There is a subset  of *SO*(3) that has measure 0 and such that if **A** is outside  then *s* = |*K*_1_|.

Proof For given **H**_*i*_ in *K*_1_ and  in *K*_2_, define the set  by

Then either (i)  or (ii)  is contained in some proper subspace of the vector space of 3 × 3 matrices. In case (i),  =  for all **W** in *SO*(3), so that  =  and therefore **H**_*i*_ **W** = **W** **H**_*i*_ for all **W** (including **W** = **I**_3_), implying that **H**_*i*_ = **I**_3_. In case (ii), there is some **C** in *SO*(3) such that tr(**CW**) = 0 for all **W** in  . Thus  has measure 0. Now let **H**_*i*_ and  run through in *K*_1_ and *K*_2_, respectively and define  as

Since this is a finite union,  has measure 0. If **H** ∈ *K*_**A**_ then **H** = **H**_*i*_ and **H** =  for some **H**_*i*_ in *K*_1_ and  in *K*_2_, so that  . Thus, if  then **H** = **I**_3_, so that |*K*_**A**_| = 1, and therefore *s* = |*K*_1_|. □

It follows from Proposition 1[Statement proposition1] that if **A** is chosen randomly from some continuous distribution then, with probability 1, *s* = |*K*_1_|.

## Appendix B Some embeddings of *SO*(3)/*K*

The embedding approach is based on embeddings, *i.e.* well defined equivariant one-to-one functions **t**_*K*_ : *SO*(3)/*K* → *E*, where *E* is an inner-product space on which *SO*(3) acts, such that **t**_*K*_([**U**]) has expectation 0 if [**U**] is uniformly distributed on *SO*(3)/*K*. Here, we focus on a simple choice of embedding **t**_*K*_ of *SO*(3)/*K* into an appropriate space of symmetric arrays, where *K* is one of the crystallographic groups *C*_1_, *C*_2_, *C*_3_, *C*_4_, *D*_2_, *D*_6_ and *O* or the tetrahedral group *T* (in Schoenflies notation). Appropriate embeddings **t**_*K*_ for general point groups *K* of the first kind are considered by Arnold & Jupp (2019 ▸) and Arnold *et al.* (2018 ▸, 2021 ▸).

It is convenient to describe the orientation of a crystal by a frame, meaning a set of vectors or axes that are fixed in the crystal. The presence of symmetry under group *K* means that such a frame is equivalent to one obtained from it by a rotation in *K*. The equivalence classes are known as *K*-frames. For *K* = *C*_1_ the frame is taken to be (**u**_1_, **u**_2_, **u**_3_) with **u**_1_, **u**_2_, **u**_3_ orthogonal unit vectors; for *K* = *C*_2_ the frame is (**u**_0_, ±**u**_1_) with **u**_0_ a unit vector and ±**u**_1_ an axis orthogonal to **u**_0_; for *K* = *D*_2_ it is a pair of orthogonal axes (±**u**_1_, ±**u**_2_); for *K* = *C*_*r*_ with *r* ≥ 3 or *K* = *D*_6_ with *r* ≥ 3, the vectors (**u**_1_,…, **u**_*r*_) are unit normals to the sides of a regular *r*-gon or hexagon; for *O* and *T*, the axes (±**u**_1_, ±**u**_2_, ±**u**_3_) and (±**u**_1_, ±**u**_2_, ±**u**_3_, ±**u**_4_) are unit normals to the sides of the cubic crystal and tetrahedral crystal, respectively. The *K*-frames will be denoted by square brackets, *e.g.* for *K* = *C*_*r*_ [**u**_1_,…, **u**_*r*_] denotes the *K*-frame arising from (**u**_1_,…, **u**_*r*_). The frames used are listed in Table 3 ▸. Note that the frames are specified entirely by vectors and axes; they do not depend on any coordinate system. For notational simplicity, we shall sometimes denote a *K*-frame by [**U**].

The embeddings **t**_*K*_ are given explicitly in Table 1 ▸. They are based on symmetric *r*-way arrays, ⊗^*r*^ **u**_*i*_, which can be thought of as *r*th powers of vectors. In mathematical terms, they are coordinate representations of *r*-fold tensor products. For vectors **u**_1_,…, **u**_*r*_ with **u**_*i*_ = (*u*_*i*,1_, *u*_*i*,2_, *u*_*i*,3_)^T^ for *i* = 1,…, *r*, the *r*-way array ⊗^*r*^ **u**_*i*_ has (*j*_1_,…, *j*_*r*_)th entry

*e.g.*  . Some of the **t**_*K*_ also involve the arrays **N**_*r*_ for *r* even. These symmetric *r* tensors are defined by

where *∥* **v** *∥* denotes the length of **v**. In coordinate terms they have entries

where Σ_*r*_ is the group of permutations of {1,…, *r*} and δ_*ij*_ = 1 for *i* = *j*, δ_*ij*_ = 0 otherwise. Thus, *e.g.*  = (  +  +  )/3. Some of the embeddings involve subtraction of constant terms to ensure that *E*{**t**_*K*_([**U**])} = 0 under uniformity. In the case of *C*_*r*_, the embeddings contain oriented vectors **u**_0_ to specify the orientation of the directed axis of symmetry.

For our purposes it is not necessary to construct the representations **t**_*K*_([**U**]) explicitly; it is sufficient to compute inner products 〈**t**_*K*_([**U**]), **t**_*K*_([**V**])〉 as required, where 〈·, ·〉 is the standard inner product on the relevant space of symmetric arrays. The inner products are listed in Table 4 ▸.

Some much more general classes of embeddings than the **t**_*K*_ given in Table 1 ▸ are considered by Hielscher & Lippert (2021 ▸) and Arnold *et al.* (2021 ▸).

In general, it is simpler and computationally faster to use inner products than to use misorientation angles. This is illustrated neatly in the cubic case. Consider [**U**] and [**V**] in *SO*(3)/*O* with **u**_1_, **u**_2_, **u**_3_ and **v**_1_, **v**_2_, **v**_3_ as the columns of **U** and **V**, respectively. Then

whereas the misorientation angle is

the minimum being over all ε_1_, ε_2_, ε_3_ = ±1 and all permutations *j*, *k*, ℓ of 1, 2, 3. Evaluating (41) involves about six times as many operations as evaluating (40).
